# S9-3 Keep up -groups support fall prevention

**DOI:** 10.1093/eurpub/ckad133.045

**Published:** 2023-09-11

**Authors:** Heli Starck, Minna Säpyskä-Nordberg, Sari Hokkanen, Jaana Viskari, Katja Borodulin

**Affiliations:** Age Institute; Age Institute; Wellbeing Services County of South Karelia; Wellbeing Services County of South Karelia; Age Institute

## Abstract

**Purpose:**

In Finland one third of home dwelling older adults 70+ fall at least once a year. Falling causes injuries and suffering. The cost of one hip fracture is about 30 000 € on the first treatment year.

Balance and functional training is effective in preventing falls. Half of hip fractures can be prevented by exercising.

**Project or policy description:**

Development: Age Institute is a partner in Age-friendly South Karelia project coordinated by Wellbeing Services County of South Karelia. The project acts through a network with municipalities, NGO’s, congregations, and older people councils. Preventing falls became an important goal of project since the number of hip fractures was increasing and higher than the average in Finland. Several actions were developed: educating professionals, giving information to older people, and improving exercise groups.

**Implementation:**

‘Keep up’ exercise group for older adults with increased risk of falling was implemented in every municipality (9) of the county. The risk of falling was assessed using a screening questionnaire in health care centers or in events, where participants with high scores were guided to the groups. Municipal sport sector organized the strength and balance training group once a week. The participants were encouraged to do home exercise weekly. The groups also included discussion and tasks about falls prevention. Themes were e.g. nutrition, fear of falling, medication and safe environment. Age Institute made the material for the group.

**Evaluation:**

SPPB test was made in the beginning and in the end of the 10 weeks training period. Majority of participants improved their physical performance. According to a questionnaire the participants perceived mostly good or strong benefits. Especially mood, strength, and knowledge of preventing falls improved. After the training period the participants were guided to continue exercising.

**Dissemination:**

The municipalities continue to organize Keep up groups. The good practice will be disseminated to other municipalities and counties involved in Strength in Old Age Programme.

**Conclusions:**

With multisectoral co-operation you can find older adults with increased risk of falling and guide them to exercise group. Strength and balance training combined with getting more knowledge is a feasible good practice.

